# Review of ‘Clean Hands Save Lives’, written by Thierry Crouzet

**DOI:** 10.1186/2047-2994-3-13

**Published:** 2014-05-05

**Authors:** Elaine Larson

**Affiliations:** 1 School of Nursing, Columbia University, 617 W. 168th St, Room 330, New York, NY 10032, USA; 2 Epidemiology Department, Mailman School of Public Health, Columbia University, 617 W. 168th St, Room 330, New York, NY 10032, USA

**Keywords:** Hand hygiene, Semmelweis

## Abstract

**Book details:**

*L’Âge d’Homme*, Tom Clegg & Thierry Crouzet L’Âge d’Homme, CP 5076, 1002 Lausanne (Suisse) 5, rue Férou, 75006 Paris (France).

## 

The concept that a ‘tipping point’ occurs when a behaviour or cultural norm reaches a threshold when sufficient knowledge, information, expectation and/or peer pressure is amassed is beautifully illustrated in this book, which tells the story of Dr. Pittet’s hand hygiene journey. Malcolm Gladwell popularized this idea in his 2000 best seller, “The Tipping Point: How Little Things Can Make A Big Difference” [1]. In his book, Mr. Gladwell uses the example of an epidemic such as measles which starts with a very few cases, but reaches a point at which the spread is rapid and daunting in its scope. Crouzet’s book has produced an easy-to-read narrative of Dr. Pittet’s tipping point contributions to global health through his work on hand hygiene.

Research demonstrating the efficacy of alcohol hand hygiene has been published around the world, starting in the 1970s and continuing through today—examples are from the UK [2-6], Croatia [7], Finland [8], The Netherlands [9], Germany [10], Austria [11-13], South Africa [14], Japan [15], and the United States [16-21]. Additionally, a hand hygiene guideline recommending alcohol-based products was first published in 1988 [22] and then again in 1995 [23], well before the CDC and WHO guidelines recommended alcoholic solutions. Hence, Dr. Pittet sits on the shoulders of others who came before him to establish a body of evidence of the efficacy of alcohol-based hand hygiene products.

But an important lesson for the scientific community is that conducting and publishing rigorous research is not sufficient for reaching that tipping point which results in changed behaviour and culture. The publication in 2002 of CDC’s hand hygiene guideline [24], for example, had only minimal impact on healthcare personnel hand hygiene behavior [25,26]. Didier took up the ‘cause’ of hand hygiene in the early 1990s and soon recognized that changing habits so engrained as hand hygiene required not only evidence of effectiveness but the application of multiple behavioural and systems-level strategies.

This book describes in colourful and rather dramatic (even evangelistic) terms the unfolding and evolution of Dr. Pittet’s work and contributions. It is not, however, a book for scientists seeking factual data. Rather it is a saga of a charismatic and committed individual who was in the right place at a tipping point and had a prepared mind and the passion to help make hand hygiene globally accessible and demonstrate its impact.

There are problems with the book. First, it contains a number of factual inaccuracies and slight misinterpretations, and, with the exception of the usual nod to Semmelweis, provides little historical perspective or knowledge of the field. Examples of some of the more egregious inaccuracies are below:

– Stating that “No one had thought of distributing it (alcohol) widely in hospitals” (p. 65) when alcohol-based products were used in a number of countries, and implying that HUG was the first place to make an inexpensive alcohol-based sanitizer in-house. In fact, a number of individuals and groups of care providers were teaching health care providers in lesser developed countries how to prepare alcohol hand hygiene preparations in their own settings. For example, in 1992 Jhpeigo, a global non-profit organization established in 1974 (http://www.jhpiego.org/content/jhpiego-history), published a manual which was widely circulated throughout many countries of the world, particularly in lesser developed regions, which not only recommended alcohol-based products for hand hygiene but also provided a ‘recipe’ for inexpensive local production [27]. The manual was again updated in 2003 [28] and has since undergone five reprintings, a testament to its widespread distribution. It offers guidance for hospitals and other facilities regarding how to institutionalize infection prevention processes such as hand hygiene. Healthcare institutions around the world were producing their own alcohol formulations for years before Dr. Pittet’s first visit to Africa.

– Attributing the ‘editorial struggle’ of the publication of one of Dr. Pittet’s early papers discussed in Chapter II to the journal’s resistance to change when, in fact, it is typical for authors to receive multiple requests for editing and revision.

– Stating that the US was purposefully failing to monitor and/or report infection rates. The author says:

“If countries such as France are exemplary in their efforts, making the use of alcohol a criterion of health care quality, others are more lax, starting with the United States. Having taken the lead in the 1970s and 1980s due to the zeal of lawyers, they then lagged behind in response to that same zeal. The best way to avoid lawsuits was to neglect measuring infection rates. Out of sight, out of mind. That meant one of the five elements of the multimodal strategy was missing: the reporting of results. All too often, U.S. health care workers do not know the real rates of infection in their service and have no means of knowing whether their performance is improving or not. In these conditions it’s difficult to correct or improve behavior” (p. 135).

Contrary to this statement, the US was one of the first countries to initiate and continue a national infection surveillance system, originally called the National Nosocomial Infection Surveillance system and continuing to current times as the National Healthcare Safety Network (http://www.cdc.gov/nhsn/). Reporting of infection rates is now mandatory in many states, albeit specific requirements differ by state, and The Centers for Medicaid and Medicare Services no longer reimburse hospitals for several potentially preventable healthcare-associated infections. Surveillance has been the cornerstone of much of CDC’s infection prevention and control efforts since the 1970s and has been continuous since then.

The author was certainly right about the fact that most US health care workers do not know the rates of infection where they work. One wonders, however, if most health care workers in France, or any other country for that matter, are privy to those rates.

The second problem with the book, from the perspective of a researcher schooled in the publishing style of peer-reviewed scientific papers, is the flowery embellishments and writing style. For example, Chapter II, “Spiritual heir to Ignaz Semmelweis” (a rather dramatic title) provides emotion-packed anecdotes about puerperal sepsis and Semmelweis’ struggles (which, in all fairness, are common in most literature regarding this important historical figure and likely to be accurate). And the dramatic description of a nurse in 1997 (!!) being booed and ridiculed by attendees at the French Society for Hospital Hygiene when she discussed using alcohol for hand hygiene is beyond belief to this US nurse researcher. The ‘Geneva model of hand hygiene’ was labeled ‘a consecration’, and Pittet’s work was likened to religious fervor and faith.

The writing style is just a personal preference, however, because the book does a great job of telling a meaningful, important, and moving story. Clearly the primary audience of this book is the lay public and the story telling style was entertaining and colourful. Just don’t use it for fact-finding! There was indeed a body of work on alcohol-based hand hygiene before Dr. Pittet began his journey, but his amazing and distinctive accomplishment has been to serve as the tipping point to globalize the importance of hand hygiene, conceptualize the practice in ways that are universally meaningful and accessible, and demonstrate its value. As Crouzet states:

“Making a gesture as simple as cleansing one’s hands into an historic innovation certainly requires a special kind of personality, a connection to people and to the most ordinary ideas, an open mind combined with an innate goodness” (p. 100).

All in all, this book accomplishes the monumental and worthy task of telling the story of hand hygiene and its role in reducing morbidity and mortality through the tipping point work of a Dr. Pittet, a visionary scientist and tireless humanitarian.
